# Case Report: Carglumic acid accelerates ammonia clearance in a neonate with methylmalonic acidemia

**DOI:** 10.3389/fped.2025.1651216

**Published:** 2025-12-01

**Authors:** Qianli Ma, Yunfeng Luo, Xu Zhang, Ling Xu, Dong Shi, Meijuan Shi, Diao Yu

**Affiliations:** 1 Department of Clinical Laboratory, Qianxi People’s Hospital, Qianxi, Guizhou, China; 2 Department of Pediatrics, Zhejiang Provincial People’s Hospital Bijie Hospital, Bijie, Guizhou, China; 3 Department of Scientific Research, Zhejiang Provincial People’s Hospital Bijie Hospital, Bijie, Guizhou, China; 4 Zunyi Medical University, Zunyi, Guizhou, China; 5 Department of Clinical Laboratory, Zhejiang Provincial People’s Hospital Bijie Hospital, Bijie, Guizhou, China

**Keywords:** carglumic acid, hyperammonemia, methylmalonic acidemia, metabolic acidosis, neonate

## Abstract

Carglumic acid, an orphan drug derived from N-acetylglutamate, activates rate-limiting enzymes in the urea cycle, thereby promoting ammonia clearance and detoxification. Carglumic acid is a potent treatment option for hyperammonemia resulting from rare urea cycle disorders; however, clinical experience and data on its use in neonatal hyperammonemia remain limited. Herein, we report the case of a 10-day-old girl with hyperammonemia secondary to methylmalonic acidemia (MMA) who experienced a resurgence of plasma ammonia levels after a reduction in dialysis replacement fluid. A sharp decrease in ammonia levels was observed on the second day following the administration of carglumic acid (200 mg/kg/day) during acute management. During a long-term follow-up of 1 year with low-dose maintenance (50 mg/kg/day) therapy, her plasma ammonia levels remained within acceptable limits, accompanied by normal neurodevelopment and growth. This case highlights that carglumic acid may be a promising therapeutic option for both acute and long-term management of hyperammonemia secondary to MMA in neonates, potentially facilitating prevention of irreversible neurological damage.

## 1 Introduction

Hyperammonemia is a common complication of neonatal organic acidemia, with approximately 61.5% of patients developing hyperammonemia (1). The mortality and disability rates can reach as high as 50%–60% if the disease is not recognized and managed promptly. Conventional methods for restricting plasma ammonia levels include dialysis and the use of scavengers (sodium benzoate and sodium phenylbutyrate). Sodium benzoate works by combining with glycine and is excreted in the form of hippurate, whereas sodium phenylbutyrate binds to glutamine to form phenylacetylglutamine, which is then eliminated from the body. Notably, sodium phenylbutyrate may exacerbate glutamine depletion, which is a key mechanism underlying hyperammonemia and energy metabolism failure in organic acids, raising concerns regarding its use (2). Although hemodialysis can effectively lower plasma ammonia levels, it may trigger hemodynamic instability and infection. Additionally, patients are susceptible to rebound hyperammonemia after discontinuation of hemodialysis. Therefore, sodium phenylbutyrate and dialysis are not ideal treatment options for patients with organic acidemia complicated by hyperammonemia.

Carglumic acid, a structural analog of N-acetylglutamic acid, was granted approval in Europe in 2003 for the treatment of inherited metabolic disorders, including N-acetylglutamate synthase deficiency and organic acidemia. Some studies have suggested the effectiveness of carglumic acid therapy in both acute and long-term management of methylmalonic acidemia (MMA) combined with hyperammonemia (3). However, approval for carglumic acid for the treatment of hyperammonemia caused by congenital deficiencies or functional impairments of urea cycle enzymes was only confirmed in June 2023 in China, and clinical experience regarding its use in treating neonatal hyperammonemia remains limited.

We report the case of a neonatal patient with hyperammonemia who was treated with long-term carglumic acid therapy, and thereby present preliminary evidence supporting its application in the management of neonatal hyperammonemia in the Chinese population.

## 2 Case presentation

A 10-day-old female infant presenting with milky vomiting and poor feeding was admitted to our hospital. She was born at 38 weeks of gestation via an uncomplicated vaginal delivery, with a birth weight of 2,459 g and Apgar scores of 10 at both 1 and 5 min. Her parents were nonconsanguineous and denied any family history of hereditary diseases or medication use during pregnancy. Physical examination revealed bilateral sluggish pupillary light reflexes and limb hypotonia.

Investigations on admission showed a blood pH of 7.29 (reference range 7.35–7.45), pCO₂ of 3.42 kPa (4.66–5.99 kPa), bicarbonate of 16 mmol/L (21–28 mmol/L), and base excess of −6.8 mmol/L (−3 to +3 mmol/L). The anion gap was elevated to 16.3 mmol/L (8–16 mmol/L), and lactic acid was markedly increased to 7 mmol/L (0.5–2.2 mmol/L). The liver and renal function tests revealed a blood glucose level of 6.9 mmol/L (3.9–6.1 mmol/L), total bile acids of 37.4 μmol/L (0–6.71 μmol/L), lactate dehydrogenase of 634 U/L (120–250 U/L), creatinine of 147 μmol/L (41–111 μmol/L), and plasma ammonia of 258 μmol/L (18–72 μmol/L; enzymatic method, AU5800, Beckman Coulter). The cardiac enzyme panel showed elevated creatine kinase [CK; 416 U/L (40–200 U/L)], CK-MB [82 U/L (0–19 U/L)], and homocysteine [19.4 μmol/L (5–15 μmol/L)]. Inflammatory markers were increased, with a C-reactive protein at 14.5 mg/L (0–6 mg/L) and procalcitonin at 0.68 ng/ml (0–0.05 ng/ml). The complete blood counts revealed white blood cells at 10.7 × 10⁹/L (15–20 × 10⁹/L), hemoglobin at 149 g/L (180–190 g/L), and platelets at 232 × 10⁹/L (183–614 × 10⁹/L). The mean corpuscular volume was slightly elevated at 103 fl (86–120 fl), and the lymphocyte percentage was high at 85.84% (26%–83%). Folate was elevated at 77.60 nmol/L (7.0–46.4 nmol/L), although vitamin B12 levels were within the normal range at 205 pg/ml (180–914 pg/ml). Urinary ketone bodies were negative. Other investigations, including stool analysis; thyroid function; levels of electrolytes, insulin, antibodies, cortisol, adrenocorticotropic hormone, and immunoglobulins; and complement and coagulation profiles, depicted normal values. Echocardiography revealed a small atrial septal defect measuring 1.9 mm. Chest and abdominal computed tomography revealed no significant abnormalities.

Given the elevated plasma ammonia levels and metabolic acidosis, organic acidemia and urea cycle disorders were suspected. In response to this diagnosis, transient cessation of amino acid intake was initiated, along with intravenous fluids constituting glucose, sodium bicarbonate (1.91 ml/kg), sodium benzoate (250 mg/kg/day), and L-carnitine (250 mg/kg/day). These measures were directed at maintaining the blood glucose at a high–normal level, reversing the metabolic acidosis, and reducing the plasma ammonia levels. Plasma amino acid and acylcarnitine profiles, urine organic acid analyses, and genetic testing were performed concurrently (at Guiyang Kingmed Diagnostics Laboratory, China).

On the second day, although metabolic acidosis showed marked improvement, the patient rapidly deteriorated into a coma with generalized convulsions. Furthermore, plasma ammonia sharply rose to 1,533 μmol/L (18–72 μmol/L). To manage this condition, the patient underwent urgent hemodialysis with a replacement fluid rate of 30 ml/kg/h. By the third day, hemodialysis had reduced ammonia levels to 98 μmol/L, which led to moderate recovery from coma and generalized convulsions. On the fourth day, however, ammonia levels rebounded to 354 μmol/L after the replacement fluid was reduced to 20 ml/kg/h. As the patient remained unconscious and comatose, precluding enteral administration, carglumic acid (200 mg/kg/day) was administered via intravenous infusion concurrent with continued dialysis. On the fifth day, the patient regained consciousness, stopped convulsing completely, and her ammonia levels decreased to 108 μmol/L. Following the clinical improvement, hemodialysis was discontinued, and carglumic acid therapy was maintained. On day seven, further metabolic testing revealed elevated plasma propionylcarnitine at 27.24 μmol/L (0.18–0.89 μmol/L, liquid chromatography-tandem mass spectrometry, SCIEX 6500, Agilent), increased plasma acetylcarnitine at 18.48 μmol/L (3.60–12.55 μmol/L), and urine methylmalonic acid at 12.7 nmol/L (0–4 nmol/L; liquid chromatography-tandem mass spectrometry, SCIEX 6500, Agilent). These findings confirmed a diagnosis of MMA. By day 10, her plasma ammonia levels had decreased to 68 μmol/L, and her neurological, mental, and nutritional status showed marked improvement. Following the positive treatment outcome, the patient's parents requested discharge. Twenty-five days later, genetic analysis confirmed the previous diagnosis. To further understand the underlying condition, whole-exome sequencing was performed on her blood-derived DNA sample (MyGenostics GenCap Core Enrichment Kit and Illumina NovaSeq 6000 platform). Bioinformatic analysis identified compound heterozygous pathogenic variants in the *MMUT* gene [c.865A>G (p.R289G) and c.1663G>A (p.A555T)]. Both these variants were classified as pathogenic according to the American College of Medical Genetics and Genomics (ACMG) guidelines.

**Figure 1:**
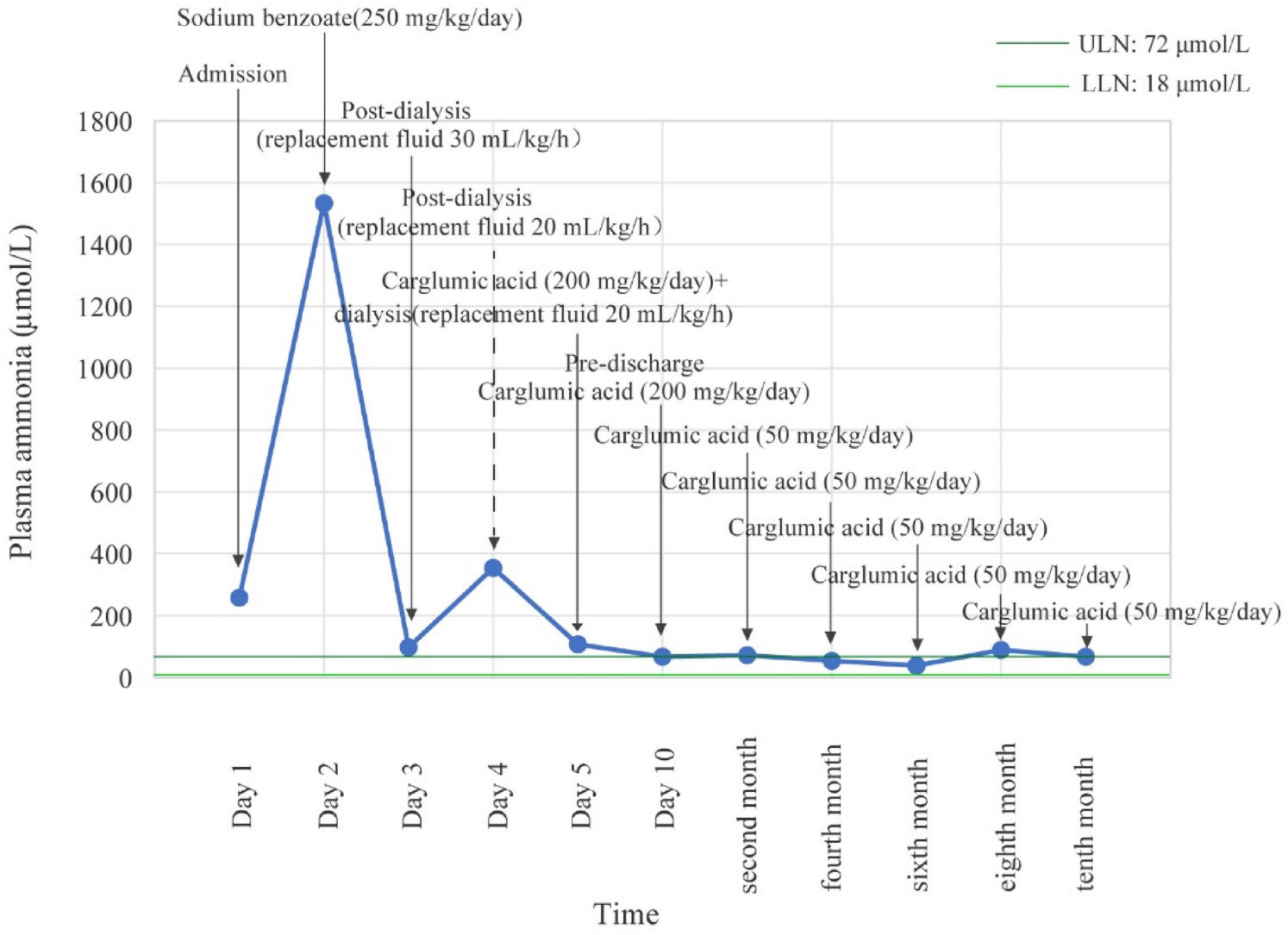
Plasma ammonia levels of the patient from admission to 1-year follow-up.

Post-discharge, nutritional management provided 1.8 g/kg/day of protein and 100 kcal/kg/day for the first 6 months, which was then reduced to 1.3 g/kg/day and 80 kcal/kg/day for the 6–12-month period. Protein was sourced from a 40:60 ratio of MMA Anamix Infant (a special formula devoid of isoleucine, methionine, valine, and threonine) to Enfamil ProSobee for the first 6 months, which was then advanced to a 30:70 ratio. Meanwhile, the patient continued to receive oral l-carnitine (150 mg/kg/day) and intramuscular vitamin B12 (1 mg/day). Carglumic acid (50 mg/kg/day, orally) was maintained as an ongoing therapy. The patient returned to the hospital every 2 months for reevaluation within 1 year after discharge. Her plasma ammonia levels corresponded to 73 μmol/L, 54 μmol/L, 39 μmol/L, 89 μmol/L, and 67 μmol/L, as illustrated in Figure 1. We evaluated her development at the 12-month follow-up using the Bayley Scales of Infant and Toddler Development, Third Edition (Bayley-III). Her scores in cognitive, motor, and language domains fell within one standard deviation of the mean, with composite scores ≥85, according to the normative data for healthy children without disease. Growth parameters (height, weight, and head circumference) and attainment of key developmental milestones were also within the normal range for her chronological age, based on World Health Organization growth standards and standard pediatric milestones. She experienced only two episodes of mild vomiting, which were not associated with the MMA episode and resolved spontaneously without any specific intervention.

## 3 Discussion

This case demonstrated that carglumic acid can swiftly decrease plasma ammonia levels during the acute management of hyperammonemia secondary to MMA. Moreover, low-dose maintenance therapy may help control plasma ammonia levels within the normal range in the long term.

Neonatal MMA with hyperammonemia presents a high risk of severe neurological injury, necessitating rapid control of plasma ammonia; however, no unified management standard currently exists. A report by Lin et al. (4) demonstrated that conventional therapy with intravenous fluids and arginine failed to prevent ammonia elevation to 2,340.2 μmol/L, ultimately requiring sustained continuous renal replacement therapy. In contrast, Tubili et al. (3) reported successful management of persistent mild hyperammonemia using carglumic acid with an initial dose of 45 mg/kg/day and maintenance dose of 20 mg/kg/day, which maintained normal ammonia levels and supported normal development. Our case presents a distinct clinical scenario of neonatal methylmalonic acidemia with hyperammonemia that responded poorly to dialysis. We implemented high dose carglumic acid (200 mg/kg/day) during acute management followed by low dose maintenance therapy (50 mg/kg/day). This approach restricted plasma ammonia within acceptable ranges and supported normal neurodevelopment and growth over 12 months of follow up. The novelty of our report lies in demonstrating the efficacy of a high dose carglumic acid regimen for acute phase treatment combined with long term maintenance therapy, supported by comprehensive ammonia monitoring and developmental assessment over 12 months. We have summarized these comparative findings in Table 1 to better contextualize our results within existing literature.

**Table 1:** Comparison of three neonates with MMA and hyperammonemia under different therapies.

Patient	Sex	Age (days)	Therapy method	Plasma ammonia (μmol/L)		Follow-up duration	Outcome	References
				Initial therapy	Final therapy			
1	Male	6	CRRT	2,340	63	41 days	Survived	Lin et al. (4)
2	Female	1	Carglumic acid (Initial dose 45 mg/kg/day, maintenance 20 mg/kg/day)	294	69	5 months	Survived	Tubili et al. (3)
3	Female	10	Carglumic acid (acute management 200 mg/kg/day, maintenance 50 mg/kg/day)	1,533	67	1 year	Survived	Our case

In an acute setting, carglumic acid has been found to rapidly lower plasma ammonia levels, which is critical for preventing irreversible neurological damage. For instance, studies from Italy and France have suggested that maintaining plasma ammonia within the normal range using the lowest effective dose of carglumic acid allows for normal growth in children with MMA, lowering the frequency of decompensation episodes and hospitalization (3, 5). Another study from the UK (6) reported the administration of 200 mg carglumic acid at 0 and 90 min, resulting in a significant reduction in plasma ammonia from 1,089 to 567 µmol/L within 90 min, which further decreased to 236 µmol/L by 6 h. Ammonia levels were normalized 12 h after two additional doses of 100 mg carglumic acid. These findings emphasize that low-dose carglumic acid may effectively sustain stable ammonia levels and improve patient outcomes.

A prospective multicenter randomized controlled trial from the Kingdom of Saudi Arabia (7) demonstrated that patients treated with carglumic acid had significantly fewer emergency room admissions due to hyperammonemia than those receiving standard care. This evidence collectively underscores the efficacy of carglumic acid in reducing plasma ammonia levels and potentially improving the prognosis of patients with MMA. The underlying mechanism is that carglumic acid enhances the activity of the rate-limiting enzyme in the urea cycle, thereby accelerating the urea cycle and intensifying the clearance of plasma ammonia from the bloodstream.

**Figure 2:**
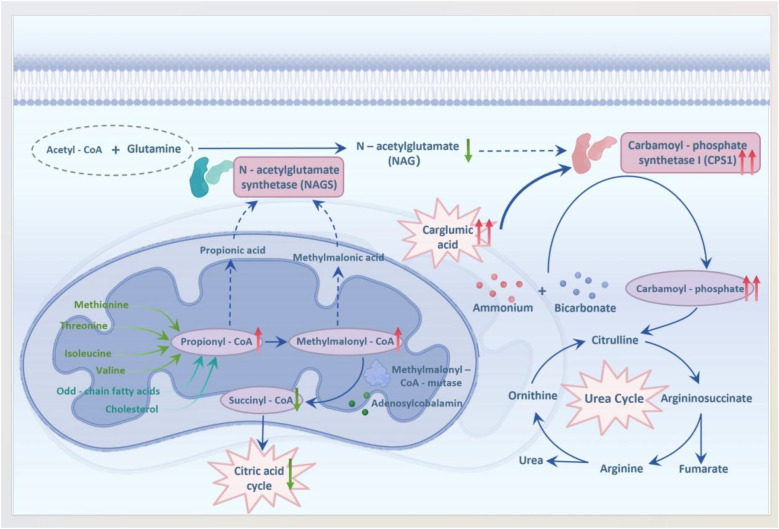
Mechanism of carglumic acid in hyperammonemia secondary to MMA (metabolite accumulation inhibits CPS1; carglumic acid restores urea cycle activity). This schematic is an adapted representation based on current understanding of the pathophysiology [References (11)].

MMA is the most common autosomal recessive disorder associated with organic acid metabolism and is characterized by multisystem involvement, including significant neurological impairment (8). MMA results from mutations affecting either the enzyme methylmalonyl-CoA mutase or its coenzyme, cobalamin. These genetic mutations disrupt the normal conversion pathway of methylmalonyl-CoA to succinyl-CoA, leading to the accumulation of methylmalonyl-CoA, propionyl-CoA, and metabolites such as 2-methylcitrate (9). Accumulated metabolites can interfere with various enzymes involved in the urea cycle by producing organic acids through bypass pathways, ultimately resulting in hyperammonemia (10). Elevated levels of methylmalonyl-CoA- and propionyl-CoA-derived metabolites inhibit carbamoyl phosphate synthase 1 (CPS1), a rate-limiting enzyme in the urea cycle that contributes to impaired ammonia detoxification. As depicted in Figure 2 (11), carglumic acid, a structural analog of N-acetylglutamic acid, mimics N-acetylglutamic acid, activating CPS1 to restore urea cycle flux and ammonia detoxification, which explains the rapid decline in plasma ammonia observed in our patient. This stimulation increases the production of carbamoyl phosphate and enhances the capacity of the body to detoxify ammonia rapidly, which is crucial during acute episodes. The rapid reduction in plasma ammonia levels achieved by carglumic acid administration underscores its potential as a targeted therapy for hyperammonemia.

This study has some limitations. First, there are no standardized dosage guidelines for carglumic acid for the treatment of hyperammonemia associated with MMA, and an optimal dosing regimen still needs to be established through further research. Second, potential adverse effects, such as recurrent vomiting, may compromise its efficacy by impairing ammonia clearance. Third, as this is a report of a single case, the findings cannot be generalized. Future research involving case series could provide more comprehensive insights.

In conclusion, carglumic acid is a promising therapeutic option for both the acute and long-term management of hyperammonemia secondary to MMA. Its ability to rapidly lower plasma ammonia levels and sustain normal levels may help prevent neurological damage and improve overall prognosis. Ultimately, larger prospective studies and case series are needed to validate our findings on the role of carglumic acid in the management of hyperammonemia secondary to MMA in neonates.

## Data Availability

The raw data supporting the conclusions of this article will be made available by the authors, without undue reservation.
